# Chalk-Stick Fracture in Ankylosing Spondylitis: A Case Report of Cervical Spine Injury Following Minor Trauma

**DOI:** 10.7759/cureus.68931

**Published:** 2024-09-08

**Authors:** Margaret L Baldwin, Erin F Shufflebarger, Zachary S Pacheco

**Affiliations:** 1 Emergency Medicine, University of Alabama at Birmingham Heersink School of Medicine, Birmingham, USA

**Keywords:** ankylosing spondylitis, cervical spine fracture, chalk-stick fracture, low-energy trauma, neurologic deficit

## Abstract

We describe the case of a 74-year-old male with ankylosing spondylitis (AS) who presented to the Emergency Department for evaluation of acutely worsened left-sided weakness following a fall from standing. Computed tomography (CT) and magnetic resonance imaging (MRI) of his cervical spine revealed a chalk-stick fracture of C5-C7, which required surgical intervention. Chalk-stick fractures are rare, typically occurring in patients with AS, and often occurring from low-energy mechanisms. In the acute setting, providers should maintain a low threshold for obtaining CT or MRI imaging to evaluate spinal injury in patients with AS.

## Introduction

Patients with ankylosing spondylitis (AS) can endure extensive spinal trauma without appreciable symptoms, as it can be difficult to assess for acute injury in the setting of their chronic disease [1]. Clinical evaluation can therefore be limited in its ability to assess for acute injury after minor trauma, making rare fractures, such as chalk-stick fractures, difficult to diagnose. This case highlights the importance of prompt imaging for the diagnosis and management of chalk-stick fractures in patients with AS. Early recognition of spinal injury can decrease morbidity and mortality in these patients [2].

## Case presentation

A 74-year-old male with a history of AS and stable left cerebellopontine angle meningioma presented to the Emergency Department with abrupt left-sided weakness and neck pain following a fall from standing. He had new difficulty ambulating due to this weakness and was unable to stand or walk on his own. The patient endorsed hitting his head but denied loss of consciousness. He also denied numbness, tingling, bowel incontinence, or bladder incontinence.

On physical exam, the patient was alert and oriented but reported severe neck pain. He was tachycardic and hypertensive with a heart rate of 110 beats per minute and blood pressure of 167/104 mm/Hg. His respiratory rate was 18 respirations per minute, and his oxygen saturation on pulse oximetry was 95% on room air. He had a Glasgow Coma Scale score of 15. A cervical collar was in place on arrival, and the patient had tenderness to palpation over the cervical spine without any appreciable step-offs, deformities, abrasions, or lacerations. On neurologic exam, pupils were equally round and reactive, external ocular movements intact, and gaze conjugate. His face was symmetric with intact strength and sensation, and his speech was clear and coherent. He had profound hearing loss at baseline that limited evaluation of coordination and pronator drift commands. His strength was 2/5 in the left upper and lower extremities and 4/5 on the right. Hoffman’s sign was positive, and he had upgoing Babinski’s bilaterally. Reflexes were 2+ and symmetric throughout.

**Figure 1:**
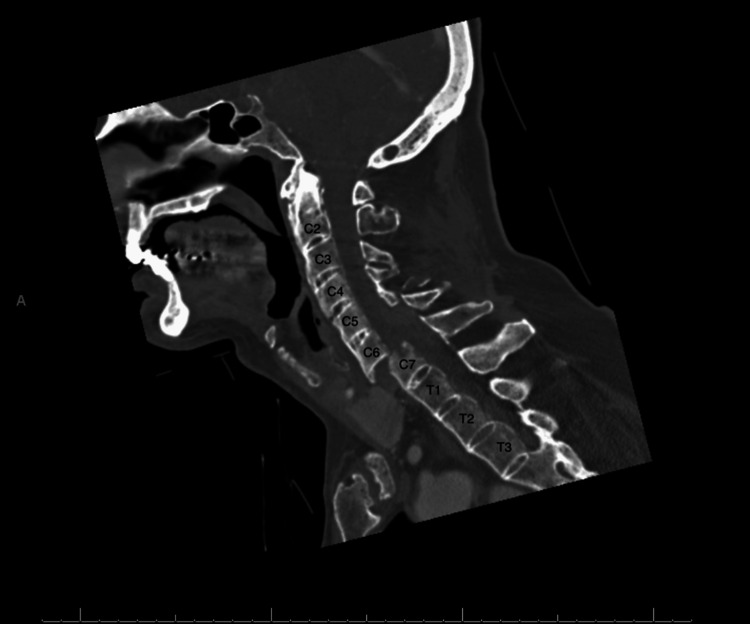
Computed tomography (CT) scan of the cervical spine showing anterolisthesis of C6 on C7 with a posterior epidural hematoma.

**Figure 2:**
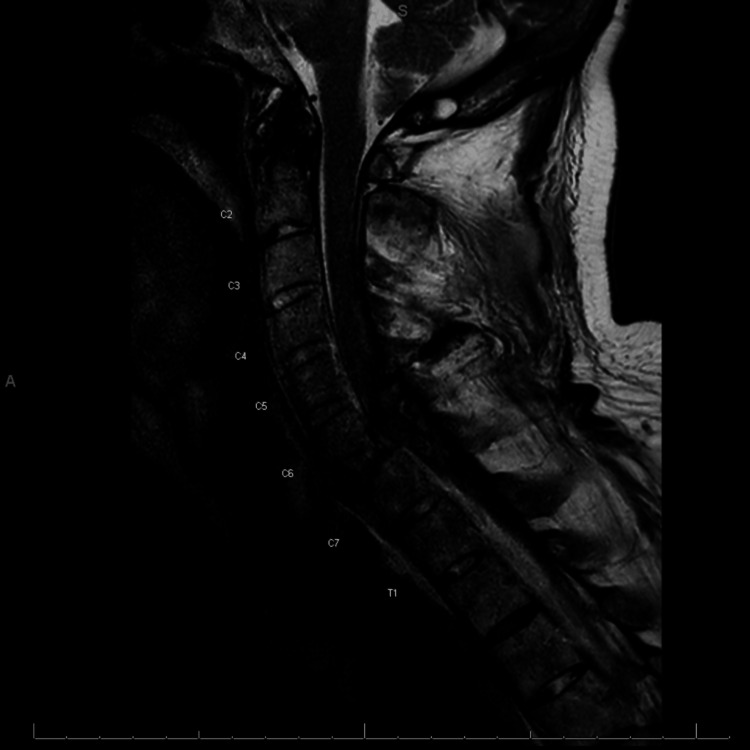
Magnetic resonance imaging (MRI) of the cervical spine showing a chalk-stick fracture from C5 to C7 with a posterior epidural hematoma.

A stroke alert was initiated pre-hospital, and the patient received non-contrasted computed tomography (CT) imaging, as well as CT angiography and CT perfusion scans of the head and neck. These studies showed his known cerebellopontine angle mass without signs of acute cerebral infarct or penumbra. CT of the cervical spine (Figure 1) revealed ankylosis of the spine, as well as a fracture through the C6-C7 disc, with involvement of the superior endplate of the C7 vertebra and right posterior inferior aspect of the C6 vertebra. There was also 75% anterolisthesis of C6 on C7 and a posterior epidural hematoma. Given these findings, orthopedic surgery and trauma surgery services were consulted. Magnetic resonance imaging (MRI) of the cervical spine (Figure 2) confirmed a chalk-stick fracture of C5, C6, and C7 vertebrae with grade 2 traumatic anterolisthesis of C6 on C7, and an epidural hemorrhage extending from C5 to the thoracic spine was again visualized.

Initial laboratory evaluation revealed mild hyperglycemia (123 mg/dL), elevated creatine kinase (291 units/L), and leukocytosis (21.54 x 10^3^ WBC with 91% neutrophils). Prothrombin time (PT) was slightly elevated at 14.7 seconds. The international normalized ratio (INR) was 1.16, partial thromboplastin time (PTT) was 28 seconds, and anti-Xa levels were less than 0.1.

The patient underwent posterior cervical decompression and fusion of C3-T3. The surgical case was completed over the course of two days due to hemodynamic instability during the procedure. Postoperatively, he remained intubated in the trauma and burn intensive care unit and was successfully extubated three days later. Due to oropharyngeal dysphagia, he required the placement of a Dobhoff tube and later a percutaneous endoscopic gastrostomy (PEG) tube. Two months after the injury, the patient was discharged home after a one-month stay in inpatient rehabilitation. At discharge, he continued to have left-sided weakness and required PEG tube feeding. After continued physical therapy sessions, the patient had improvement in his left-sided weakness at his outpatient follow-up one month later.

## Discussion

This case involves a rare spine fracture from a low-energy mechanism of injury. As prompt diagnosis and intervention significantly impact outcomes, providers should maintain a high level of suspicion for spinal injury in patients with underlying AS [2].

This patient was at increased risk for his chalk-stick fracture due to underlying spinal osteoporosis that is associated with AS [3]. Chalk-stick fractures, also known as carrot-stick fractures, typically occur in patients with AS from a flexion-distraction injury [4]. These fractures involve a complete horizontal fracture of the vertebrae, intervertebral disc, and other spinal segments [1,5]. The cervical region, specifically C6-C7, is most often affected, and these fractures often occur from low-energy mechanisms, like the fall from standing experienced by this patient [1,6]. A majority of AS patients present with a neurologic deficit, but due to difficulties differentiating their chronic disease from a new event, diagnosis is frequently delayed by patients waiting to seek care and initial imaging failing to reveal the acute event [1,7]. Additionally, the National Emergency X-Radiography Utilization Study (NEXUS) criteria and Canadian C-spine rule, which are common clinical decision rules for pursuing imaging in cervical spine trauma, either do not address or specifically exclude their use in patients with underlying AS [8,9]. Providers should instead consider spinal injury in a patient with AS when there is a history of any direct or indirect trauma to the spine, a complaint of spinal pain aggravated by activity and relieved by rest, or any change in posture [3]. Suspected spinal injury should then be swiftly evaluated by obtaining a CT or MRI, with literature suggesting that a CT is sufficient in identifying acute spine injury in the majority of cases. MRI may be the better imaging modality when investigating pathology within the spinal canal and disco-ligamentous hyperextension injuries [10].

Management of a stable chalk-stick fracture may be conservative, with cervical collar placement aimed at restoring the patient’s preinjury alignment. Surgical intervention is considered in the case of deteriorating neurologic status, irreducible deformity, and the presence of an epidural hematoma or other source of spinal cord compression [1]. Of note, between 0.5% and 1.7% of chalk-stick fractures may result in spinal epidural hematoma, and delayed hematoma occurring hours after injury has been reported [4,11,12]. In this patient, surgical intervention was necessary due to the instability of his fracture and the presence of epidural hematoma. The decision for conservative versus surgical management should be made with assistance from a surgical consultant. Therefore, the role of emergency medicine and other acute care providers is to make a prompt diagnosis of spinal injury using CT or MRI, as delay in diagnosis can lead to devastating neurologic consequences [6].

## Conclusions

Emergency physicians should have a low threshold to evaluate spinal trauma in patients with AS, even when the injury is from low-energy mechanisms. Advanced imaging, such as CT or MRI, should be utilized expeditiously, as prompt diagnosis can reduce morbidity and mortality.
